# Culture, social networks and HIV vulnerability among men who have sex with men in Indonesia

**DOI:** 10.1371/journal.pone.0178736

**Published:** 2017-06-05

**Authors:** Nelsensius Klau Fauk, Maria Silvia Merry, Mitra Andhini Sigilipoe, Sukma Putra, Lillian Mwanri

**Affiliations:** 1 Institute of Resource Governance and Social Change, Jl. R. W. Monginsidi II, Kel. Kelapa Lima, Kec. Kelapa Lima, Kupang, Nusa Tenggara Timur, Indonesia; 2 Medicine Faculty, Duta Wacana Christian University, Jl. Doktor Wahidin Sudiro Husodo, Yogyakarta, Indonesia; 3 Bina Nusantara University International, Jl. Hang Lekir I, Senayan, Jakarta, Indonesia; 4 Discipline of Public Health, School of Health Sciences, Flinders University, Adelaide, South Australia, Australia; Oregon State University, UNITED STATES

## Abstract

The current study aimed to explore cultural and social network influence on HIV vulnerability among Men who have Sex with Men (MSM) population in Yogyakarta, Indonesia. A qualitative inquiry employing in-depth one-on-one interviews was carried out with 24 MSM participants in July 2015. Data were analysed using a framework analysis and guided by the Social Networks Theory (SNT) as a conceptual framework. Findings indicated that prohibitive cultural perspectives and norms against same-sex marriage made them to conceal their sexual orientation and thus secretively engaging in unprotected sex that increased their predisposition to HIV transmission. The prohibitive cultures were also instrumental in the formation of MSM sexual networks that provided supportive environment for HIV-risky sexual practices among network partners. These findings provide information that can be used to improve HIV/AIDS service practices and policies. However, further studies with large numbers of MSM would be needed to improve the understanding of other HIV vulnerability determinants, the unique needs of MSM, and what and how programs could be conducted to reduce HIV vulnerability among MSM population.

## Introduction

Over the past three decades, HIV infection has become a significant global public health problem, and Indonesia has not been immune. The 2016 world AIDS report showed that an estimated 36.7 million people worldwide have been infected with HIV, of whom 95% live in developing countries [1]. In Indonesia, the numbers of HIV cases have continuously increased during the last few years [2, 3]. This increase has been attributed to annual increases in new numbers of HIV infections [2]. For example, HIV infections increased from 21,032 cases in 2011 to 21,511 in 2012, 29,037 in 2013, 32,711 in 2014, slightly decreased to 30,935 in 2015, and increased to 41,250 in 2016 [2]. Of 237,641,326 Indonesian population, there have been 319,103 HIV/AIDS positive people, comprising 232,323 people with HIV infection and 86,780 people with AIDS [2, 4].

The mode of HIV transmission in Indonesia is mainly through sexual contact [2, 5]. Available report indicates that for the past five years the infection was highly prevalent among sexually active age groups, including 71.0% among people aged 25 to 49 years and 15.4% among those aged 20 to 24 [2]. Men are reported to be more vulnerable to the infection than women since the first diagnosis of the epidemic in September 1987 [2, 6]. In the last five years, the percentage of HIV positive men was higher than that of women, representing 59% and 41% respectively [2].

Estimates indicate that the population of Men who have Sex with Men (MSM) in Indonesia is three million, a population recognised to be the most at high risk for HIV infection in the country [2, 5, 7, 8]. During the recent years, the diagnosis of new infections in MSM has steadily increased every year, with cases reported as follows during the past seven years: 506 cases in 2010, 1040 cases in 2011, 1514 cases in 2012, 3287 cases in 2013, 3858 cases in 2014, 4241 cases in 2015, and 13,063 in 2016 [2]. In the Special Region of Yogyakarta where this study was conducted, the current data have reported that of the total of 4,648 HIV/AIDS cases, 616 were diagnosed in MSM [9].

A number of determinants associated with the vulnerability of MSM to HIV infections [10–15] have been reported in various studies including: (i) having unprotected sexual intercourse with multiple casual sex partners, (ii) engaging in transactional sex, (iii) using illicit drugs and excessive alcohol, and (iv) finding casual sex partners through internet for offline sex [12–15]. The lack of knowledge about the means of HIV transmission and prevention among MSM population has also been described as one of the factors leading to MSM’s engagement in unprotected anal intercourse (UAI) and injecting drug use (IDU), and enhancing the HIV transmission [11, 16–18]. In some cultural groups such as within African American and Latinos, family rejection or being evicted from home due to sexual orientation have also been reported to play a key role in enhancing MSM’s vulnerability to HIV infections [19–21]. As a result of this rejection, individuals attain new social life situations that facilitate their engagement in risky sexual behaviours including having casual and unprotected sex with new partners [19].

In Indonesia, the community cultural perspectives and norms do not allow the recognition/acceptance of MSM, and may also play a role in increasing MSM’s vulnerability to HIV infections [21]. Studies by Budiman [22] and Boellstorff [23] have indicated that being a gay in Indonesia is seen as both a sexual deviance and contamination towards Indonesian culture. Moreover, if a person deviates from the dominant heterosexual norm, that person may be regarded not only as sick (having psychiatric problem) and/or sinful, but also not a proper Indonesian citizen [23, 24]. Such community perspectives and norms have often led to MSM stigma, discrimination, judgemental behaviours, rejection, and threatened violence [21, 25, 26]. These have also led to MSM’s reluctance to discuss sexual health problems with significant others such as family members or health professionals [27]. This reluctance has led to MSM lacking information or limiting them to accessing HIV/AIDS-related services such as voluntary counselling and testing (VCT) and condoms [26]. The lack of discussion of such problems, and of access to the services often supports their engagement in unprotected sexual behaviours hence increasing their vulnerability to HIV transmission [18, 28–30]. Additionally, the lack of recognition/acceptability of MSM status in the Indonesian general community cultural perspectives and norms, and in the national Laws, and the forbiddance of gay activities and events or congresses promoting gay rights in Indonesia have contributed to unfavourable environment for social interactions among MSM [22–25, 31]. Such drastic conditions may force MSM to be more supportive of gay friendly environments and the formation of a solid gay community. Here, the community is defined as a group of people living in the same place or having a particular common characteristic or a feeling of fellowship with others, as a result of sharing common attitudes, interests and goals. Such a community facilitates the formation of social network defined as a network of social interactions and personal relationships among MSM [32, 33]. Social influences among MSM peers which have been reported to provide opportunities such as linking casual sex partners within the community have also been reported to enhance MSM’s engagement in unprotected sexual encounters [10].

To our knowledge, no evidence is available in regards to cultural and social network determinants of MSM vulnerability to HIV transmission in the Indonesian context. Unprotected sexual behaviours with multiple sex partners, injecting drug use and sexually transmitted infections prevalence have been reported to be determinants of MSM vulnerability to HIV transmission among this population in the country [5, 7, 8]. The aim of this study was to explore the influence of cultural and social network on vulnerability of MSM population to HIV transmission in Yogyakarta, Indonesia.

## Methodological approaches

### Study setting

The Yogyakarta (Daerah Istimewa Yogyakarta or DIY) region is one of the 34 provinces in Indonesia and consists of 1 municipality, 4 districts, 78 sub-districts, and 438 villages [34]. Located in the South of Java Island, Yogyakarta covers an area of 3,158.80 km^2^, bordering the Central Java province and the Indian Ocean [34]. With the population density of 1,084 people per km^2^, the province is reported to have 3,452,390 people, comprising 1,705,404 men and 1,746,986 women [35]. Yogyakarta city, the only municipality in the province and the current study focal setting is divided into 14 sub-districts and 45 villages [34. It is inhabited by 636,660 people with the population density of 13,340 people per km^2^ [35]. The majority of people in Yogyakarta are cultural groups, including Javanese (the main group comprising about 87% of total population), Sundanese, Malay, Chinese, and Batak [35]. It has 2 government hospitals and 18 private hospitals, 18 public health centres and 9 sub-public health centres [36].

### Theoretical framework

The Social Networks Theory (SNT) [32] was employed as a heuristic tool to guide the conceptualisation and the analysis of this exploratory study. The SNT analyses and informs how cultural perspectives and norms can shape social networks and influence behaviours, and hence health outcomes of groups and populations. We have used SNT because Asian culture (including Indonesia) is based on collectivist characteristics/behaviours, where the idea of the community as a collectivist influences actions in the entire and/or part of the community [37, 38]. The Indonesian communal way of life and social networking brings together people forming ‘a community,’ that is heavily involved in communal events [37, 38]. These community networks and dynamics are very important because they provide identity, social support, social influence, person-to-person contact and access to resources that can have impacts on individuals’ health outcomes [32, 37]. Similarly, cultural perspectives and norms of same-sex relationships and marriages contribute to shaping MSM social network structure variable in range or size (the number of MSM members in the network), density (to what extent MSM are connected to each other), and homogeneity (to what extent individuals are similar to each other in the network). Cultural perspectives and norms can also contribute to shaping characteristics of network ties such as frequency of contact among MSM (number of face-to-face contact), multiplicity (number of types of transaction among MSM), duration (the length of time MSM know each other) and intimacy among MSM [32]. These social networks’ structures and characteristics also provide opportunities for social interactions and influences among MSM through connecting casual sex partners, supporting engagement in unsafe/unprotected anal intercourse, and concealment of sexual orientation, hence increasing their vulnerability to HIV acquisition/transmission [32, 39, 40].

### Study design

The study employed a qualitative inquiry using in-depth one-on-one interviews [41]. This design was deemed to be appropriate to explore participants’ own values, meanings and interpretations regarding their behaviours of lived experiences and social relationships, and how these positioned them in HIV transmission [42–44]. The qualitative design has also been described to be effectives in studying people’s perspectives in their natural settings [42], hence providing a deep insight of their real life experiences [42, 45, 46].

### Recruitment

Study participants were MSM in Yogyakarta, Indonesia. Participants were recruited through a purposive sampling method because the study aimed at a specific research objective. The initial participant who was an NGO staff and worked with MSM population was contacted, and he agreed to participate in the study. Upon being requested, he provided information to his friends who accepted to be contacted by the researchers, and upon being contacted they participated in the research. Each participant was contacted via mobile phone. A total of 24 participants were enrolled in the study. They knew and interacted with each other a few times a week and were also in personal relationships either as friends or lovers. They were parts of broader MSM community and social network in Yogyakarta. The inclusion criteria for the selection of participants were: (i) aged 18 years and above, and (ii) identified themselves as MSM. Characteristics and demographic information of the participant are described in findings section.

### Procedure

Data were collected in July 2015 by researchers (NKF,MSM and MAS) using in-depth one-on-one interviews [42]. Underpinned by Social Network Theory (SNT), interviews explored cultural perspectives and norms on same-sex sex relationship and marriage, and how such perspectives and norms contribute to shaping social networks among MSM population. Additionally, we explored the role that social networks play in providing social support, social influence, person-to-person contact and access to resources.

### Ethical considerations

The research was conducted in accordance with human research conventions that include confidentiality, voluntariness and informed consent. Prior to the interviews, participants were informed about the aim of the study and that their participation was voluntary and there would be no consequences if they decided not to participate. They were also informed that the interview would take approximately 45 to 90 minutes, and would be audio recorded using a tape recorder. To inform the participants about the research, a written information sheet detailing the aim and nature of the research was provided before participants were asked to sign a consent form. After reading the information sheet, each participant signed a written consent form prior to the interview. Participants were assured of the anonymity and confidentiality of the collected information. To maintain individuals’ anonymity and obscure their identity, each participant was assigned a study identification letter and number (e.g. R1, R2,….). Interview with each participant was scheduled, and took place at their convenient time and places recommended by the participants. On the interview day, each participant was given a light snack and IDR.75,000 (~€5) to reimburse for their time to participate and travel to interview. The interviews were conducted in Bahasa. The study Ethics approval was obtained from the Medicine Research Ethics Committee, Duta Wacana Christian University, Yogyakarta, Indonesia (Ref. No.: 002-B/C.10/FK/UKDW/VI/2015).

### Data analysis

Data were transcribed verbatim and translated into English by (NKF) and verified by (MSM and MAS). These authors speak Bahasa and English fluently, making it easy to verify for accuracy and the quality of translation. To maintain the reliability and validity of the collected data, data were crossed checks for accuracy and clarity among the three authors during the transcription and translation process. The analysis used Ritchie and Spencer’s framework analysis [47], involving five steps. The first step involved *familiarisation* with the data or transcripts by reading them line by line repeatedly, breaking down into several chunks of data, and giving comments or labels. The second step included *identifying a thematic framework* where recurrent key issues, concepts and themes were written down. Informed by the Social Networks Theory, a thematic framework or coding frame was identified and data coding scheme developed. The third step involved *indexing* the entire data. A list of open codes was analysed looking for similar codes and redundant codes. This helped to reduce the list to a smaller and manageable number for further analysis. This was followed by creating closed coding where codes referring to the same theme were grouped together. This process took several stages until a short list of 5 overarching themes was reached. They were (i) cultural perspectives and norms on same-sex marriage, (ii) development of stronger social networks among the gay community, (iii) desire to conceal sexual orientation, (iv) the influence of living environment, and (v) HIV vulnerability among MSM population. The fourth step involved *charting* the data through arrangement of appropriate thematic references in a summary chart so that it could be compared across the interviews and within each interview. The Fifth and final step was *mapping and interpretation* to examine the ideas that made up the main themes in order to see the relationship and association between them [32, 47]. It is acknowledged that the framework provides a systematic approach to the management of qualitative data in a coherent and structured way. The framework also enhances rigour, transparency and validity of the analytic process [47].

## Findings

### Characteristics of the participants

The median age of the participants was 30.5 years. All participants lived in Yogyakarta city and had moved there from other Indonesian provinces during the past five years. Participants belong to 6 different ethnicities including Java, Bali, Makassar, Banjar, Atoni and Sumbawa. Their education level varied as follow: the majority (66.6%) graduated from high school, 29.2% were university students and 4.2% had a bachelor degree. Similarly, their employments differed with 58.3% and 12.5% working as entrepreneurs and NGO staffers respectively. About a third (29.2%) of participants were unemployed. Most participants reported to have been diagnosed with at least one of the following sexually transmissible infections (STI): HIV, syphilis, gonorrhoea, and chlamydia. Five participants reported to know their HIV status as being positive.

### Cultural perspectives and norms on same-sex marriage

Prohibitive cultural perspectives and norms against same-sex sexual orientation and marriages seemed to have played a strong role in enhancing participants’ vulnerability to HIV infections. This resulted in negative outcomes including making them to conceal their MSM status and missing necessary interventions against HIV prevention:

“My parents and sisters do not know that I am a gay. I cannot tell them because I am afraid that they will get disappointed. I am afraid that they are not ready yet to accept my status because they live in a rural area and firmly hold the norms that are against same-sex marriage, and are religious as well. …. This situation also made it difficult for me to talk to my sisters or friends about safe sex or condoms, or VCT like now” (R22: 21 years old).

“I have never been open to anybody in my family about this [his sexual orientation]…. I often think of telling them about myself [sexual orientation] but I am worried whether they can accept it or not……. Before moving here I was afraid of talking to other people about sexual health, I did not know anything related to sexual health” (R19: 32 years old).

“I do not tell my family members and other people [about his status as a gay] in the community where I lived [his place of origin] because I am afraid of being rejected.… I did not talk to anybody about my sexual life, I kept everything on my own. Now I do realise that I was at high-risk for HIV infection due to my own behaviour [unprotected sex]. I was just lucky that I did not get infected [with HIV]” (R8: 23 years old).

In Indonesia, it is culturally expected that men would marry women, and at their ages, participants were expected to have married or revealed to their significant others, the potential spouses. In their circumstances, the stress of being repeatedly asked whether they were in love with a girl, or when they would get married, made them decide to find freedom away from home, and where they could engage in casual relationships with multiple sex partners:

“I feel depressed living there [place of origin], depressed with the questions: who is your girlfriend? When will you get married? If I continue living in my place of origin I will become someone else, lie to myself and to people, and pretend to be a normal person [heterosexual]. It is a big pressure because many friends of mine have got married and have kids. So I decided to move here [Yogyakarta] so that I can meet people [MSM] who are in the same group as me.… Here, I can do what I want with my [sex] partners without being worried too much about the judgements or views of people surrounding me” (R19: 32 years old).

“Moving here [Yogyakarta] at least prevents me from the pressure from my mom who always encourages me to get married as soon as possible. I like it here because we [MSM] are more welcome, and it is easy for me to find [sex] partners.” (R24: 29 years old).

“To me, it is better not to live with my parents and older sisters because I do not need to answer their question about whether or not I am in love with a girl. Now I am living far away from them so I can lie to them that I already have [a girlfriend] but I do not have to introduce her to them because they are not here.… Yes, I do have sex partner and even more than one but they all are men, hahahaha” (R13: 25 years old).

### Development of stronger social networks among the gay community

As a coping strategy to deal with Indonesian cultural perspectives and norms against MSM orientation, participants moved to Yogyakarta and developed new or engaged in the existing MSM social networks. They also connected with networks of social interactions and personal relationships among the broader gay community known as *komunitas sehati* (one heart community). These networks and interactions afforded comfort and continuity to further MSM relationships. These assertions are supported by the following:

“We [MSM] have our own [gay] community called *Komunitas Sehati* and regularly meet each other a few times a week. I feel comfortable to talk or share my personal information with them [MSM friends] because I know they are at the same position as me, and we [MSM] often have similar experiences and problems.… You know in our country we are the ones being rejected, none of the cultural norms in any parts of this country accepts our sexual orientation and allows same-sex marriage. So our [MSM] social relationships are somewhat limited among us [MSM]” (R7: 27 years old).

“I am close to many other friends [MSM] here in Jogja [nick name for Yogyakarta] because we meet very often. Most of us [MSM] are connected to each other through one heart community and I think most of us in this community know each other as friends or boyfriends [lovers]. I think the reason why we [MSM] stick to each other in our [one heart] community because we feel that most [heterosexual] people see us differently, as weird people, at least I feel that. This might be due to the socio-cultural norms and values that support heterosexual marriage and reject same-sex sexual relationship” (R1: 22 years old).

“My close friends are from the one heart community.… Maybe because we [MSM] understand each other better than the others [heterosexual people] understand us. This is the reason why I trust my MSM friends more than non-MSM friends” (R21: 34 years old).

Participants indicated that the gay community was significantly large, a few thousand people. They acknowledged to have been involved in MSM networks or parts of the social interactions among MSM in Yogyakarta. It was also acknowledged that the number of MSM in the networks increased over time because new MSM from many other cities and provinces across Indonesia were coming or moving to Yogyakarta:

“I do not know the exact number of the total MSM population in Jogja but so far our [HIV/AIDS] program has reached more than two thousand MSM, and there are many other we do not cover yet. Not all of them who have participated in our program are actively involved in MSM social interactions or routinely come and meet others at the gay events or common spots but I can tell you that there are quite a lot who are very active. And I am sure the number of MSM here increases over time because there are always new faces joining us” (R14: 25 years old).

“I think there are thousands of gay people in Jogja and many of us especially the ones who are actively involved in gay activities or events or come to gay spots during the weekend know each other. We can easily get to know each other through our social networks…. I have known my colleagues [other MSM] for a few years because I moved here since four years ago, some I have known for about 2 to 4 years but some I just met a few months ago……” (R20: 27 years old).

It is obvious that social networks provide opportunities for social interactions and influence among MSM, including providing the supportive environment for connecting casual sex partners and supporting engagement in unprotected anal sexual practices with multiple casual sex partners. These activities increasingly enhance their vulnerability to HIV infections:

“We [MSM] meet each other every week, two to three times during the week days and at the weekend at the gay spots or clubs, so we know each other quite well… Every time we meet we talk and share information about many things including health, condoms, sex partners and so on. Some of my [casual] partners were at first introduced by my other [MSM] colleagues at the gay spots.” (R7: 27 years old).

“…. I often introduce my [MSM] friends who do not have [sex] partners to each other when we get together. Some of my [sex} partners were also introduced by my friends.…. So far I do not use condoms when having sex with my casual partners because they are just short-term partners, after one or two or three times having sex then we leave each other” (R4: 26 years old).

“I have sex with multiple concurrent sex partners but we [he and his partners] never talked about safety [condom use] every time we had sex because we do not have commitment to having long-term relationships. All of them are my casual [sex] partners whom I met at the gay spots or introduced by other [MSM] friends.… Many other friends also told me the same story, they do not talk about condom use with their casual partners” (R18: 24 years old).

### Desire to conceal sexual orientation

It was evident that MSM tried to conceal their sexual orientation from those who were not a part of their social networks including medical professionals such as nurses, doctors and others in health service settings. As the consequence, MSM self-medicated including for sexually transmissible infections (STIs) and obtained medication from their fellow MSM:

“I felt ashamed to undergo medical check up at the community health centre because at that time I got inflammation on my anus.…. It would be very difficult for me explain if a doctor or nurse asks how I got it. I was afraid because they would know about my sexual orientation” (R12: 22 years old).

Peer influence and lack of information about how to access sexual health services seemed to be the factors supportive of the concealment of MSM’s sexual orientation from the medical professionals and poor health seeking behaviour. However, those who were lucky to know the HIV/AIDS program run by Vesta, a non-governmental organisation providing HIV/AIDS services for MSM community in Yogyakarta, were able to open up when they visited this health service:

“I once got an infection; at first, I did not know what that was. I was very afraid because I felt painful when I peed. I was also afraid to visit general practitioner because I did not know what to say, so I told a close friend of mine about that and he suggested me to buy antibiotics he previously took because he said the symptoms were similar to what he got.… but it recurred for three times so at last I attended VCT provided by Vesta and I was diagnosed with gonorrhoea” (R2: 25 years old).

“I remember I got the infection when I was just a few months moving here [Yogyakarta]. I did not know yet about Vesta and its HIV/AIDS program. The only way I could do was talking to my friends [MSM] and they gave me antibiotics.….” (R8: 23 years old).

### The influence of living environment

Supportive environment including the availability of meeting points for MSM community provided them with avenues to interact which often led to unsafe casual sex practices. All the participants interviewed acknowledged that there were a few public places (‘hot spots’) in Yogyakarta that had become the meeting points for MSM including bars, cafes, malls, night clubs, and city parks:

“We [MSM] very often get together such as at *Alun-alun Utara* [North square], Malioboro mall, *Taman Pintar* [city park], and several cafes and night clubs. These are also the places for us to look for [sex] partners. For example, if I meet a partner [in these places] and we feel attracted to each other, then we can go back home and execute [have sex]” (R18: 24 years old).

“There are a few places where we [MSM] meet but *Alun-alun Utara* is the well known one, even others [MSM] from other cities come to this place. We also have our own gay night events at night clubs where we get to know each other, and look for sex partners. I met a few of my [casual sex] partners at night clubs” (R1: 22 years old).

Public places were also common spots used by MSM to engage in sex transactions (sell or buy sex) which involved unprotected and unsafe sex. Such environment was believed to play an important role in increasing MSMS social networks, and enhanced the vulnerability of MSM population to HIV infections:

“Some of my colleagues [MSM prostitutes] and I usually wait for clients at *Taman Pintar* and *Alun-alun Utara*, and sometimes in Malioboro Mall. Clients come and choose, and then bargain. If the price is okay, then it [sex intercourse] happens. Everybody [MSM] knows this kind of thing” (R16: 23 years old).

“I often go to *Taman Pintar*, the East part where a bookstore is located is also a place where we [MSM] meet. That is where there are many [MSM] sex workers in the evening.…. I also sell [sex] at this place [*Taman Pintar*], if I get client then we go to cheap hotel, check in and execute [have sex]” (R17: 28 years old).

The living environment supportive of MSM social interactions and personal relationships was another reason why the participants chose to move to Yogyakarta. They acknowledged that Yogyakarta is an environmentally gay friendly place where they were not discriminated or abused in public and had spaces for social interactions with other MSM:

“Jogja is a nice place for me to live. I have never been verbally abused since I moved here a few years ago. It is not the same as my place of origin. I can freely go everywhere around Jogja without any hesitation of getting abused” (R9: 27 years old).

“Here in Jogja people do not discriminate me. I think people recognise that I am a gay but do not act discriminatively against me. I once read that the governor has also asked people here to respect us, and this might explain why it is environmentally gay friendly here” (R11: 22 years old).

### HIV vulnerability among MSM population

Vulnerability to HIV infection seemed high among the MSM population in Yogyakarta. This was acknowledged by all the study participants because they were aware of the fact that many of their MSM colleagues had previously been diagnosed with the infection.

“I feel that I can get the [HIV] infection if I am not careful enough because some friends of mine are now living with it. I think they got from their [MSM] partners” (R23: 28 years old).

“I am an HIV positive person and I decided to work for the [Vesta] organisation because I want to help other [MSM] colleagues. I know there are other MSM who have the same [HIV] status as me and many other MSM are also vulnerable to acquiring the infection because of unprotected sexual behaviour” (R10: 32 years old).

“Hundreds of MSM in Jogja have been infected with HIV and they are highly likely to transmit it to their partners because I am sure the majority of us [MSM] are inconsistent in condom use. So, I think MSM are vulnerable to the infection, I am vulnerable [to the infection] as well” (R5: 42 years old).

## Discussion

Studies conducted in many different countries and settings have reported various factors associated with the vulnerability of MSM population to HIV infections [12–16]. Findings of the current study inform that cultural perspectives and norms that do not allow same-sex marriage are prohibitive and lead to concealment of the sexual orientation by MSM, predisposing them to further sexually transmissible infections. Similarly, cultural expectations of heterosexual marriages seemed to exacerbate HIV transmission in the participants. Because of stigma and fear of rejection by significant others, MSM were unable to discuss sexual health-issues with family members, friends or health professionals. At times, participants would avoid health care service provision and self medicate, leading to further disadvantages and poor health outcome. While these findings seem to be particular to these settings, they are not dissimilar to reports from previous studies [18, 28, 29]. In addition to concealing their sexual orientation and avoiding discussions about health issues or attendance to health care provision, these findings also suggest that cultural norms and perspectives against same-sex relationship or marriage espoused their decision to move to Yogyakarta where they developed and/or engaged in MSM social networks. In other words, such perspectives and norms contributed to shaping the structure and characteristics of MSM’s social networks, and supporting their engagement in HIV-risky sexual behaviours. Because in Yogyakarta they are tolerated and not judged, they socialise easily and engage in casual sex with multiple partners, exacerbating their vulnerability to STIs including HIV [48, 49]. These findings are in line with the previous findings [19, 20], where the rejection by family members due to sexual orientation has been reported to increase the likelihood of having unsafe sex that favours the spread of HIV infection among MSM.

Consistent with previous studies [10, 50–52], these findings reaffirm the influence that social networks among MSM have in increasing the likelihood of MSM’s engagement in unprotected casual sex with multiple partners, and increasing their vulnerability to HIV infections. Torres and colleagues [10] have also shown that peer influence contribute to increased MSM’s involvement in unprotected anal intercourse. Similar to findings in previous studies [53–57], in the current study, peers had multiple roles in influencing behaviours that enhanced participants’ vulnerability to HIV infections, including providing supportive environment for MSM to have sexual contacts, and supporting self-medication when unwell. Similar to findings of research conducted elsewhere [53–56], in the current study, supportive physical environment, such as the availability of gay meeting point was one of facilitating factors for participants’ social interactions, including supporting their engagement in transactional sex and unprotected sexual behaviours, hence increasing their vulnerability to HIV transmission.

### Limitations of the study

There are several limitations that need to be addressed in the current study. First, the study involved small numbers of samples and the majority of the participants were from the same site, the city centre of Yogyakarta. It is therefore less likely to generalise the study results to other MSM populations in Indonesia and other similar settings. Second, the fact that all the study participants were recruited from the same site might have resulted in under-sampling of MSM from other parts of Yogyakarta. Such under-sampling might result in an incomplete or biased overview of the influence of culture and social network on HIV vulnerability among MSM populations. The study also did not explore the relationship between ‘being a gay’ and an ‘Indonesian national identity’ and its influence on MSM cultures and behaviours. These are important issues that could be explored in future studies.

## Conclusions

The current study generates knowledge on how cultural norms and perspectives about same-sex sexual relationship and marriage contribute to shaping social network structures and the characteristics of network ties among MSM. Furthermore, these structures and ties provide opportunities for social interactions and that enhance vulnerability of MSM population to HIV infections. Although only five (21%) of the current study participants reported that they were HIV positive, it is plausible to affirm that they are overrepresented in HIV data, when compared to the overall Indonesian population HIV prevalence, which is only about 0.13%. Understanding the influence that social networks and social influence have on enhancing the vulnerability of MSM to HIV infections in the study setting is important in order to develop effective strategies to halt the vulnerability to the HIV scourge among MSM and the general population. This understanding can also be used to inform HIV/AIDS programs to take into account the needs of MSM [58], such as free condoms and lubricants provision [59, 60], MSM-friendly voluntary counselling, HIV voluntary testing and other services [60, 61], and to include peer-led interventions with the aim of reaching as many as MSM population [62–65]. Such targeted programs have been proven effective in increasing risk perceptions and knowledge of HIV/AIDS, and condom use among MSM population in six Asian cities [58, 59]. Although the current study’s findings are very informative, further studies to understand what could be done by the government and nongovernmental organisations at local and national level to reduce HIV vulnerability among MSM population are recommended.

### Directions future research

Given the current state of knowledge of HIV vulnerability determinants among MSM populations, a few areas for future studies canbe identified. First of all, more studies with MSM communities need to be carried out to explore other HIV vulnerability determinants that were not covered in the current study. Secondly, it is also necessary to conduct multisite studies which could enrol large numbers of MSM because the samples in the current study as well several previous studies were relatively small. Given the socio-cultural context of Indonesia which is prohibitive of same-sex sexual relationship or marriage that makes the recruitment of MSM population is a challenge, coverage of large numbers of participants would be possibly reached through multisite data collection. This would not only increase the size of study sample but also variability of sample, and reduce sampling bias. Thirdly, studies focusing on identifying the unique needs of MSM populations and exploring the relationship between ‘being a gay’ and an ‘Indonesian national identity’ would provide some insight as to how this relationship influences MSM cultures and behaviours in Indonesian MSM populations.
